# A SNP assay for assessing diversity in immune genes in the honey bee (*Apis mellifera* L.)

**DOI:** 10.1038/s41598-021-94833-x

**Published:** 2021-07-28

**Authors:** Dora Henriques, Ana R. Lopes, Nor Chejanovsky, Anne Dalmon, Mariano Higes, Clara Jabal-Uriel, Yves Le Conte, Maritza Reyes-Carreño, Victoria Soroker, Raquel Martín-Hernández, M. Alice Pinto

**Affiliations:** 1 Centro de Investigação de Montanha, Instituto Politécnico de Bragança, Campus de Santa Apolónia, 5300-253 Bragança, Portugal; 2 Agricultural Research Organization, The Volcani Center, Rishon LeTsiyon, Israel; 3 INRAE, Unité Abeilles et Environnement, Avignon, France; 4 IRIAF, Instituto Regional de Investigación y Desarrollo Agroalimentario y Forestal, Laboratorio de Patología Apícola, Centro de Investigación Apícola y Agroambiental (CIAPA), Consejería de Agricultura de la Junta de Comunidades de Castilla-La Mancha, Marchamalo, Spain; 5 Instituto de Recursos Humanos para la Ciencia y la Tecnología (INCRECYT-FEDER), Fundación Parque Científico y Tecnológico de Castilla—La Mancha, 02006 Albacete, Spain

**Keywords:** Biological techniques, Genetics

## Abstract

With a growing number of parasites and pathogens experiencing large-scale range expansions, monitoring diversity in immune genes of host populations has never been so important because it can inform on the adaptive potential to resist the invaders. Population surveys of immune genes are becoming common in many organisms, yet they are missing in the honey bee (*Apis mellifera* L.), a key managed pollinator species that has been severely affected by biological invasions. To fill the gap, here we identified single nucleotide polymorphisms (SNPs) in a wide range of honey bee immune genes and developed a medium-density assay targeting a subset of these genes. Using a discovery panel of 123 whole-genomes, representing seven *A. mellifera* subspecies and three evolutionary lineages, 180 immune genes were scanned for SNPs in exons, introns (< 4 bp from exons), 3’ and 5´UTR, and < 1 kb upstream of the transcription start site. After application of multiple filtering criteria and validation, the final medium-density assay combines 91 quality-proved functional SNPs marking 89 innate immune genes and these can be readily typed using the high-sample-throughput iPLEX MassARRAY system. This medium-density-SNP assay was applied to 156 samples from four countries and the admixture analysis clustered the samples according to their lineage and subspecies, suggesting that honey bee ancestry can be delineated from functional variation. In addition to allowing analysis of immunogenetic variation, this newly-developed SNP assay can be used for inferring genetic structure and admixture in the honey bee.

## Introduction

The worldwide movements of humans and goods, coupled with climate change, have led to the introduction, and often successful spread, of many pathogens and parasites into novel environments, and this phenomenon is occurring at unprecedented temporal and spatial scales^1^. The range and host shifts resulting from these introductions are threatening many organisms across the globe, from mammals^2,3^, birds^4^ amphibians^5^, to fishes^6^. Within insects, the honey bee *Apis mellifera* L. has been particularly impacted by introduced parasites and pathogens, most notably the mite *Varroa destructor *and the microsporidian *Nosema ceranae*^7–10^. These are both native to Asia and have rapidly spread worldwide, after a host shift from *Apis cerana* to *Apis mellifera*^7,10^. *V. destructor* suppresses the bee immunity, but most importantly it acts as a reservoir, incubator and transmission route for several viruses^11^, including one of the prime honey bee pathogens: the Deformed Wing Virus (DWV). *N. ceranae* is an intracellular parasite, which decreases colony longevity by inducing oxidative stress and by causing changes in metabolism and immune response^8,9,12^.

With emerging diseases becoming major selective pressures, colony survival will ultimately depend on how honey bees are able to successfully activate immune mechanisms to protect themselves against foreign pathogens, at both colony (social immunity) and individual (individual immunity) levels. Social immunity arises from behavioural cooperation and includes removal of adult corpses (necrophoric behaviour), removal of diseased or parasitized larvae (hygienic behaviour), and over-production of heat (thermoregulatory behaviour; reviewed by Evans and Spivak^13^ and DeGrandi-Hoffman and Chen^14^). Individual immunity entails different lines of defence, ranging from physical barriers (e.g. exoskeleton cuticle and peritrophic membranes lining the digestive tract) to cellular (e.g. phagocytosis, nodulation, encapsulation mediated by hemocytes, and melanization of hemolymph catalysed by phenoloxidase) and humoral responses (synthesis and secretion of antimicrobial peptides, AMPs, such as abaecin, hymenoptaecin, apidaecin, and defensin^14–16^. In addition to cellular and humoral responses, RNA interference (RNAi) has been described as a major mechanism of antiviral defence in honey bees^14,17–19^.

The honey bee immune system is triggered when structural motifs on the surface of pathogens (pathogens-associated molecular patterns—PAMPs) bind to its receptors (pattern recognition receptors—PRRs) activating different pathways, depending on the pathogen. These pathways include Toll, IMD (immune deficiency), Jak-STAT (Janus kinase and signal transducer and activator of transcription), and c-Jun N-terminal kinase (JNK). The honey bee has fewer immune genes than *Drosophila melanogaster*, *Aedes aegypti*, or *Anopheles gambiae*^17,20^. Nonetheless, all the main components of the major pathways, as well as the AMPs, identified in these Dipteran model species are shared by the honey bee^17^.

Although immune genes are highly conserved across species, some of them also display substantial intra-specific genetic variation^21–24^. Different studies show that this level of variation affects disease resistance in a wide range of taxa, from mammals^25,26^ to insects^22,24,27^. In fact, there is evidence that a small number of large-effect genetic variants has a substantial contribution to differential susceptibility to many diseases in humans^28^ as well as in arthropods^27,29^. In honey bees, studies on immune response to infection have been conducted at the gene-expression level for important pathogens and parasites (e.g.^11,30–33^). However, the link of these findings to inter-individual variability at the DNA level is lacking.

Single nucleotide polymorphisms (SNPs) have been widely used to screen genetic diversity in immune genes (e.g.^21,23,33–36^) and to identify genes associated with susceptibility to pathogens and parasites in many organisms^2,23,36,37^ but not in honey bees. As a first step towards filling this gap, here we developed a medium-density-SNP assay for genotyping immune genes in the honey bee. This assay contains 91 quality-proved SNPs located in functional regions of 89 immune genes and these can be readily genotyped with the high-sample-throughput iPLEX MassARRAY system. To the best of our knowledge, this is the first medium-density assay tailored for genotyping SNPs involved in immune response in honey bees.

## Results

### Assay design, quality control and genotyping accuracy

**Figure 1:**
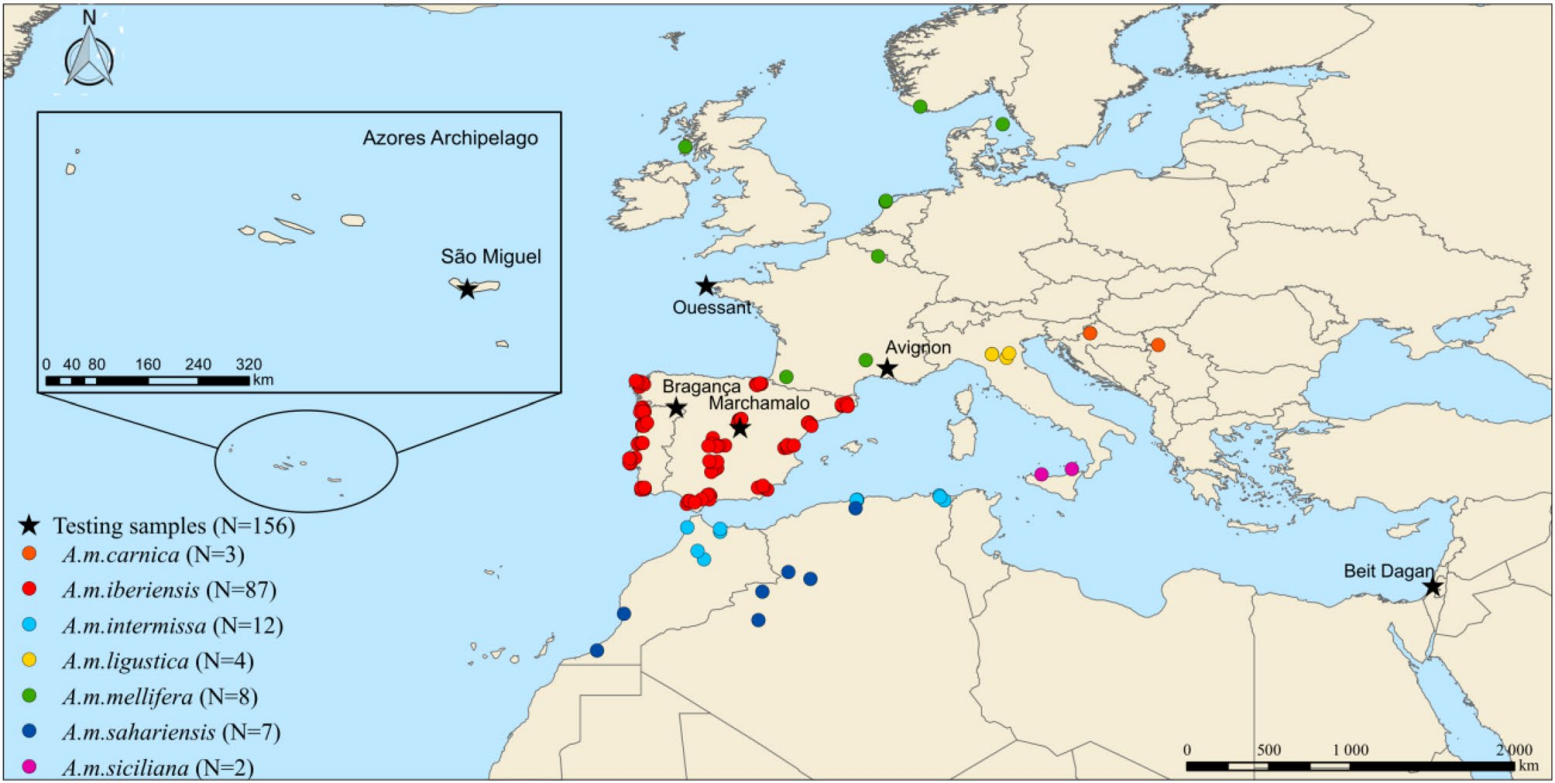
Geographic locations of the 123 whole-genome samples (represented by dots) used for constructing the SNP assay and of the 156 independent samples (represented by stars) used for testing (map generated by QGIS 3.12.2- București- https://qgis.org/es/site/forusers/download.html).

A total of 209 immune genes were compiled from the literature review, but 29 could not be found in the reference genome Amel_4.5. The remaining 180 genes were scanned for polymorphisms in the 123 WGs (Fig. 1; Supplementary Table S1).

These genes are marked by 35,782 SNPs of which 20,308 are intronic and 11,602 intergenic (Supplementary Table S2). The genes containing the highest number of SNPs are GB46759 (3486) and GB49918 (3032), both related with antiviral defence^17^. Of the initial 35,782 SNPs, only 4938 could be assigned to putative functional regions, including 2876 (2307 synonymous; 569 non-synonymous) in protein coding, 36 in intronic < 4 bp from exons, 1030 in intergenic < 1 Kb upstream of the transcription start site, 183 in 5’ UTR, and 813 in 3’ UTR. These functional SNPs cover 177 genes, with GB50482 (serine protease) and GB55483 (immunoglobulin-like and fibronectin type III domain) genes harbouring the highest numbers with 233 and 399 SNPs, respectively (Supplementary Table S2).

**Figure 2:**
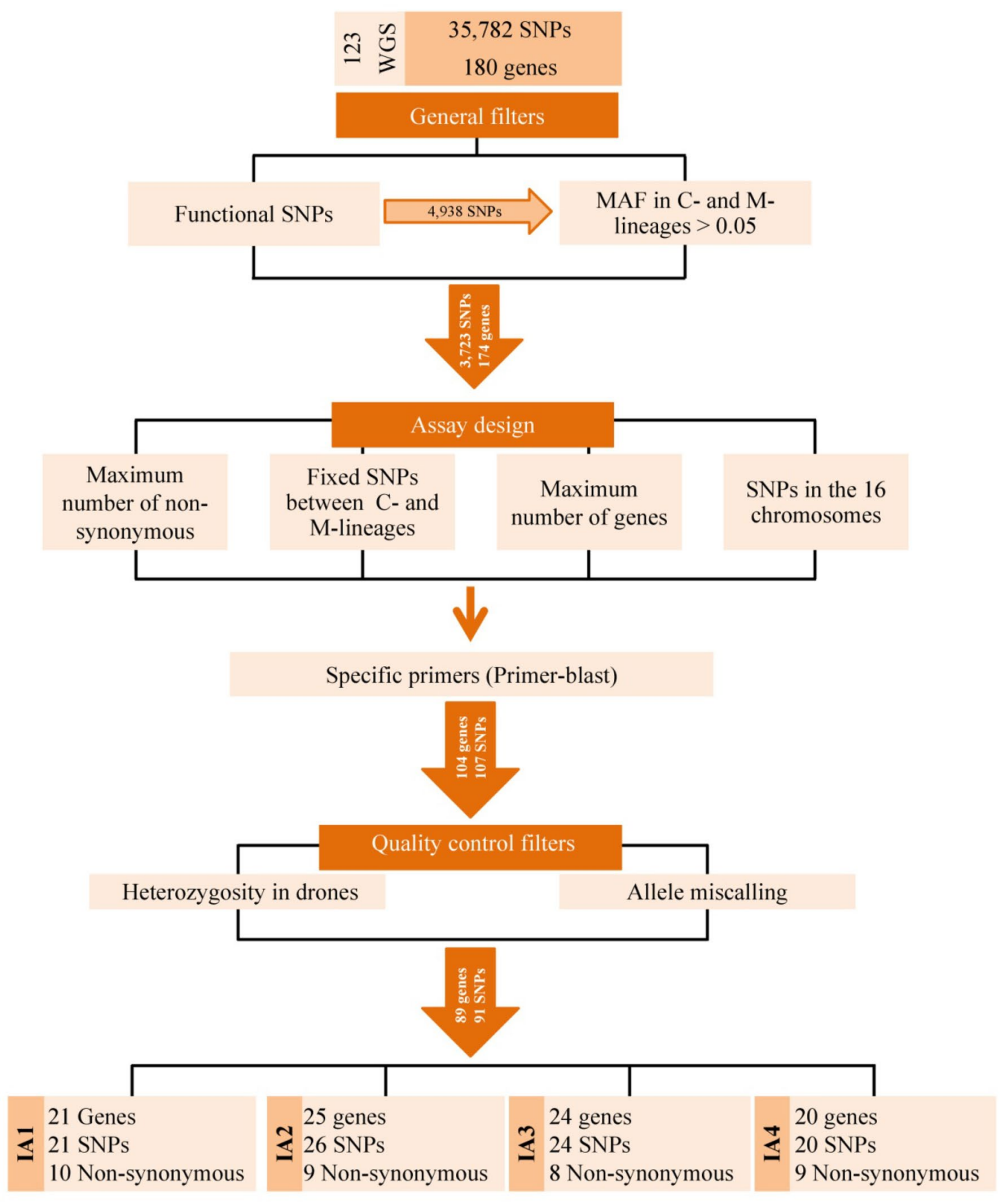
Diagram depicting the different criteria and steps involved in the development of the four SNP assays (IA1, IA2, IA3, and IA4) using as a baseline whole‐genome sequence data from 123 individuals representing seven honey bee subspecies.

**Figure 3:**
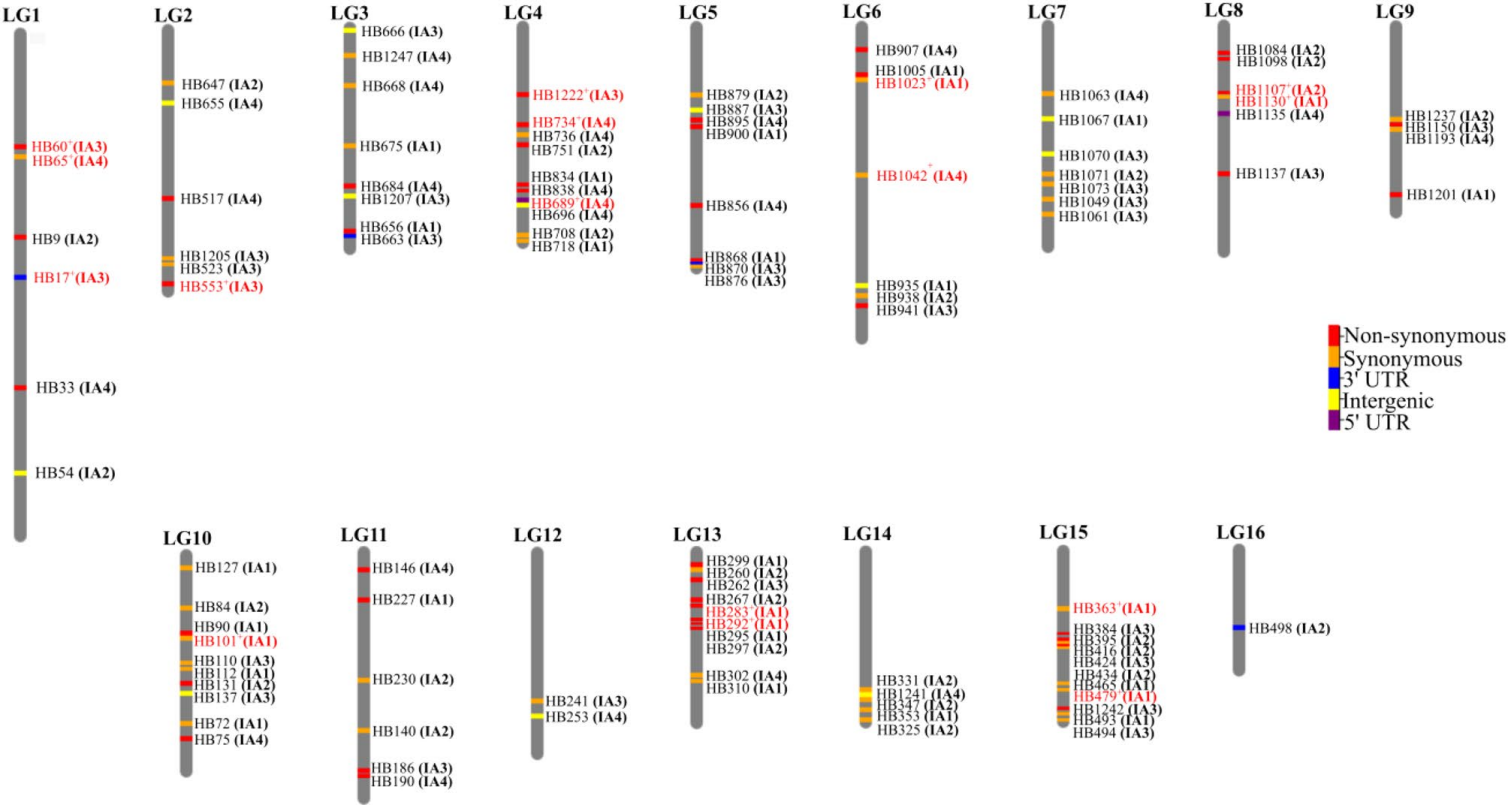
Chromosome map showing the SNP positions in the four assays (IA1–IA4). Each functional state is represented by a different colour. SNPs coloured in red were discarded by at least one filtering criterion.

Among the 4938 functional SNPs only those that revealed to be polymorphic (MAF > 0.05) within the M- and C- European lineages were retained, allowing further downsizing to 3723 SNPs. In the subsequent filtering step, which was linked to the MassARRAY MALDI-TOF genotyping system, the flanking regions of the 3723 SNPs were used as input in the AssayDesign software. Despite the high number of possible SNP combinations, none of the final designed multiplexes reached the desired 30-plex size, due to predicted hairpin and dimmer formation artefacts, with IA1 and IA3 containing 28 SNPs, IA2 27, and IA4 only 24 (Fig. 2). Altogether, the four assays allow genotyping 107 SNPs, of which 44 (41%), 61 (57%) and 72 (67%) were polymorphic for the C- (*A. m. carnica* and *A. m. ligustica*), M- (*A. m. mellifera* and *A. m. iberiensis*) and A-lineages (*A. m. intermissa* and *A. m. sahariensis*), respectively, and 46 (43%) were fixed (F_ST_ = 1) between C- and M- lineages (Supplementary Table S3). These SNPs cover 104 immune genes distributed across the 16 honey bee chromosomes (Fig. 3).Genomic information for the 107 SNPs as well as their flanking sequences and the PCR and iPLEX reaction primers, for genotyping in the MassARRAY MALDI-TOF platform, are shown in Supplementary Table S4.

The SNP calling quality and genotyping accuracy obtained for the four assays with the iPLEX MassARRAY system was assessed in 18 haploid individuals that have been sequenced for the whole-genome in the HiSeq 2500 platform^41,42^. This endeavour was greatly facilitated by screening haploid drones because we could reliably detect false heterozygous loci. Of the total 1926 called genotypes (107 SNPs × 18 individuals), 131 (6.8%) revealed to be inaccurate (Table 1). The most common source of mismatch between the two platforms was due to heterozygous positions erroneously called in > 10% of the haploid individuals by the MassARRAY system (75 genotypes). However, most mismatches (96%) could be assigned to only 14 SNP loci. In addition to this problem, three SNPs (HB1107, HB60, HB734) exhibited inconsistent allele calls between the two platforms in > 10% of the samples. One of them, locus HB734, was also prone to be misidentified as heterozygous in the haploid individuals (Supplementary Table S5). After removing the 16 problematic loci, we ended up with 91 quality-proved SNPs distributed as follows: 21 in assay IA1, 26 in IA2, 24 in IA3, and 20 in IA4 (Fig. 2; Supplementary Table S4).

**Table 1:** SNP calling quality assessed for the 107 SNPs genotyped in 18 haploid individuals (honey bee males) using the iPLEX MassARRAY system. Calls were compared with those obtained with the Illumina HiSeq 2500 platform for the same individuals.

SNP calling	Number of genotypes
Miscalling—inconsistent alleles	22
Miscalling—heterozygosity	75
Missing data	34
Accurate calls	1795
Total	1926

**Figure 4:**
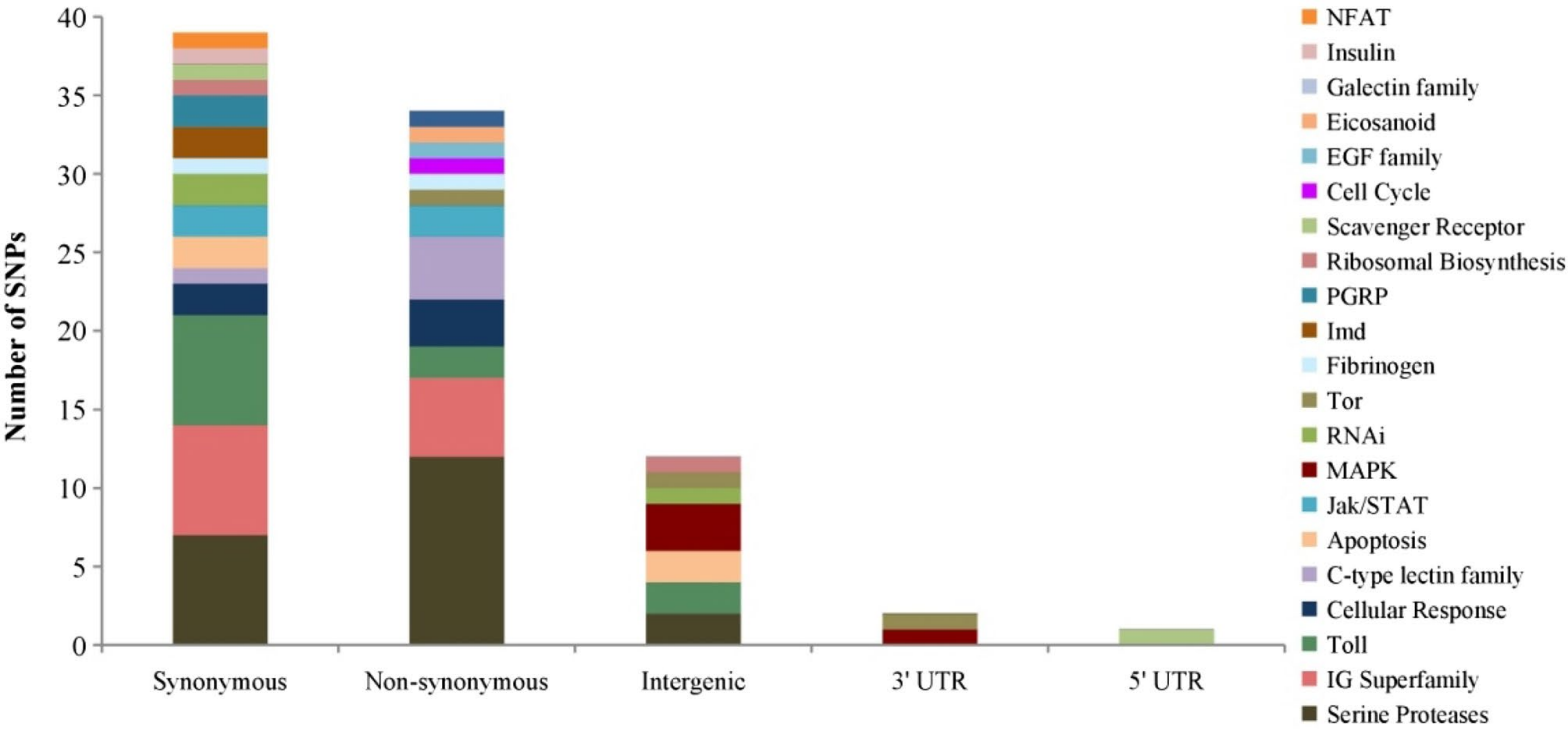
Distribution of the 91 SNPs by functional state and pathway/group.

The 91 SNPs cover 89 immune genes that belong to a wide array of pathways and groups, being serine proteases the most representative (Fig. 4; Supplementary Table S4). Important immune-related pathways are marked by more than one SNP, including IMD, RNAi, JAK/STAT, and Toll, with 2, 3, 4, and 11 SNPs, respectively. Twenty three of the 89 genes have been found to be differentially expressed in honey bees infected with different viruses and *Nosema* spp. (Table 2; Supplementary Table S4). The 91 SNPs are putatively functional, with the great majority (75) laying in protein coding regions (39 synonymous and 36 non-synonymous). While sixteen SNPs are non-coding, they are likely of functional relevance as they are located in 5’ UTR (1), 3’ UTR (3) and in < 1 Kb upstream of the transcription start site regions (12), where the promoter is expected to be located^21^.

**Table 2:** SNPs mapped to genes differentially expressed in honey bees infected by different pathogens.

SNP	Gene	Pathway	Pathogens	References
HB663	GB41227	Undescribed	SINV	^17,43^
HB656	GB41241	Undescribed	SINV	^17,43^
HB1241	GB41669	Apoptosis	Nosema spp.	^33^
HB1237	GB42706	Apoptosis	*Nosema apis*	^33^
HB1067	GB43303	Ribosomal biosynthesis	IAPV	^17,44^
HB1061	GB44311	Ribosomal biosynthesis	IAPV	^17,44^
HB186	GB45248	Undescribed	IAPV	^17,39^
HB868	GB45735	Endocytosis	IAPV	^17,31^
HB498	GB47575	MAPK	IAPV	^17,31^
HB302	GB47804	PGRP	DWV; IAPV	^17,39,40^
HB310	GB47805	PGRP	DWV; IAPV	^17,39,40^
HB1207	GB49154	Apoptosis	Nosema spp.	^33^
HB1063	GB49244	Endocytosis	IAPV	^17,31^
HB465	GB50020	Jak/STAT	IAPV	^17,31^
HB1242	GB50261	Cell cycle	*Nosema ceranae*	^33^
HB494	GB50290	Undescribed	IAPV	^17,39^
HB736	GB50418	Toll/TLR	IAPV	^17,39^
HB395	GB50508	Undescribed	SINV	^17,43^
HB72	GB50955	RNAi	IAPV; SINV	^17,39,43^
HB75	GB51047	Undescribed	*Nosema ceranae*	^45^
HB253	GB52015	MAPK	IAPV	^17,31^
HB137	GB52625	MAPK	IAPV	^17,31^
HB1205	GB55605	Apoptosis	*Nosema apis*	^33^

### Applying the SNP assay

The 91 SNPs were genotyped on pools of workers collected from 156 colonies from France (Avignon and the island of Ouessant), Israel (Beit Dagan), Portugal (Bragança and the island of São Miguel) and Spain (Marchamalo, Fig. 1). The quality control of the MassARRAY-generated genotypes indicates a high genotyping success rate of the 156 samples. Missing data per locus ranged from 0% (36 SNPs) to 21.8%, although only two SNPs (HB1049 and HB895) exceeded 15% (Supplementary Table S5). Missing data per individual was also low, varying between 0 and 12.1%, with most individuals (83.3%) below 5% (Supplementary Table S6).

**Figure 5:**
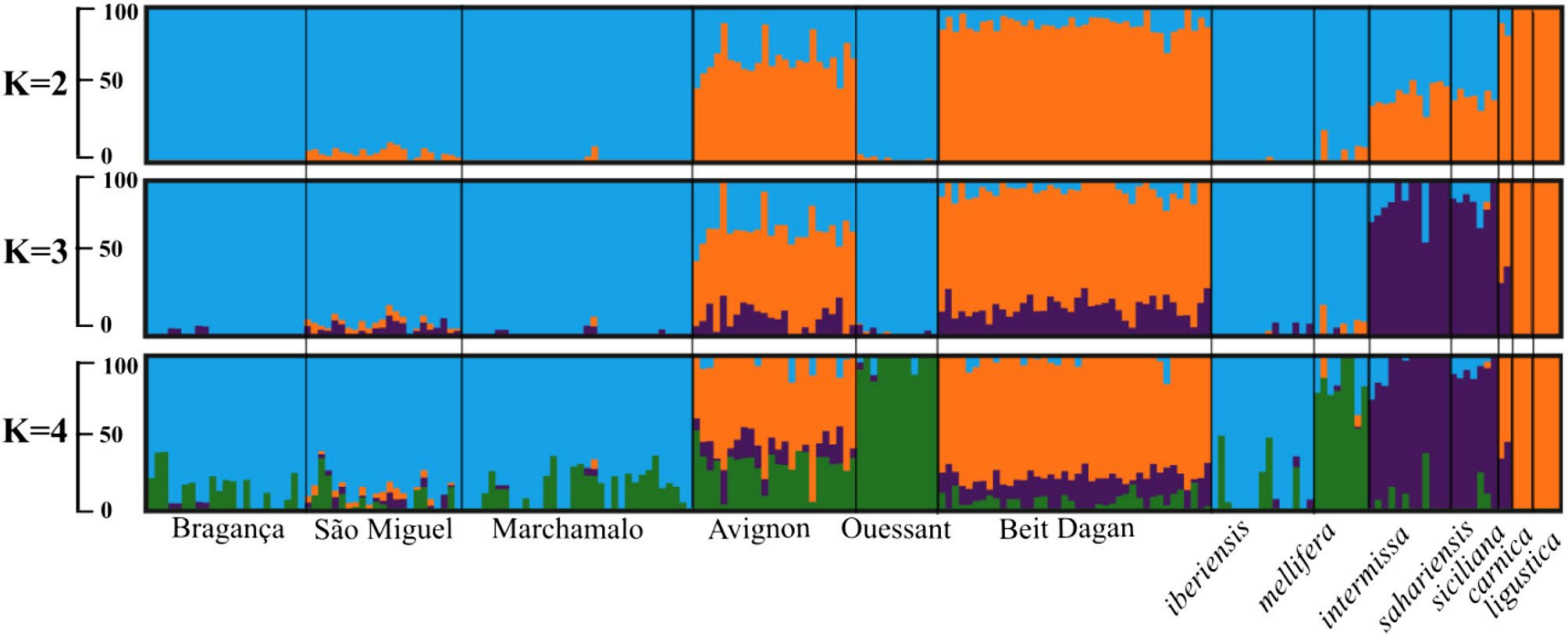
Admixture plots showing the membership partitioning (Q-value) from two (K = 2) to four clusters (K = 4). Each individual is represented by a vertical bar. The black vertical lines separate individuals from different populations (Bragança and São Miguel-Portugal; Marchamalo-Spain; Avignon and Ouessant- France; Beit Dagan- Israel).

The MAF distribution for each SNP and population is shown in Supplementary Fig. S1. Locus HB938 is the least polymorphic (MAF = 0.05) whereas HB310 is the most polymorphic, with both alleles displaying equal frequencies (MAF = 0.5). For 75.8% of the SNPs, the greatest contrast in allelic distribution is exhibited between Beit Dagan plus Avignon and the remaining populations (Ouessant, Bragança, São Miguel, Marchamalo). This contrasting pattern is further reflected by membership proportions (Q-values) inferred by ADMIXTURE for one to seven K genetic groups. According to the cross-validation error, the most likely K is three (Supplementary Fig. S2), although it is only at K = 4 that the two M-lineage subspecies (*A. m. mellifera* and *A. m. iberiensis*) split (Fig. 5). At K = 4, populations from Avignon (average Q-value = 0.575, Supplementary Table S6) and Beit Dagan (average Q-value = 0.774, Supplementary Table S6) share an important genetic component with the eastern European *A. m. carnica* and *A. m. ligustica* reference subspecies (orange cluster). This suggests that both populations are mostly of C-lineage commercial ancestry, although they also have a background assigned to the clusters of the reference subspecies *A. m. mellifera* (green cluster) and the north African *A. m. intermissa* and *A. m. sahariensis* subspecies (purple cluster). On the other hand, the remaining populations show negligible levels of C-lineage ancestry and they are assigned to clusters that match the historical distributional ranges of the subspecies, i.e. samples from Portugal and Spain are assigned mostly to the *A. m. iberiensis* cluster (blue) and samples from Ouessant (France) are assigned to the *A. m. mellifera* cluster (green).

## Discussion

In the last decades, international trade in honey bees and their products has facilitated global dissemination of exotic parasites and pathogens^46,47^, many of which became major causes of colony losses^7,10,48^. In addition to be a vehicle for disseminating parasites and pathogens at the global scale, trade can also help exotic gene flow within the *A. mellifera* natural range, leading to introgressive hybridization or even displacement of native subspecies^49–51^. In previous studies, we developed SNP-based tools for estimating introgression of C-derived commercial honey bee stocks into M-lineage subspecies from Western Europe^42,52,53^. In this study, we expanded the existing SNP-tool box by developing a medium-density assay for screening polymorphisms in immune genes. Of note is that, in addition to allowing surveys of putatively functional variation in 89 immune genes^14,17,30,33,38,45,54–56^, this assay can discriminate subspecies belonging to different evolutionary lineages and detect C-derived introgression in colonies with diverse genetic background.

Development of the medium-density assay begun with detection of SNPs across the honey bee immunome. The entire coding regions of 180 genes (< 1 kb upstream of the transcription start site, exons, introns < 4 bp from exons, and UTRs) were scanned on 123 whole genomes, representing seven subspecies and three evolutionary lineages (A, M, and C). By sampling such an important fraction of the *A. mellifera* diversity, this discovery panel enabled detection of SNPs with low frequency of the minor allele (average MAF = 0.19), therefore minimizing ascertainment bias^57^. With the exception of the recently published study of Momeni, et al.^58^, this is the largest and broadest panel used for SNP discovery in the honey bee. Previous works screened narrower discovery panels, suited to the goals being addressed by the constructed assays. While Momeni, et al.^58^ screened 14 subspecies to construct a ~ 4 K SNP assay for diagnosing European subspecies, others used discovery panels with less than four subspecies to construct high- to medium-density SNP assays tailored for addressing multiple objectives, including analysis of variation linked to defensive behaviour against *V. destructor*^59,60^ genomic selection and breeding^60^, identification of Africanised honey bees^61,62^, and inference of C-lineage introgression into M-lineage subspecies^42,52,63^.

Selection of SNPs for the medium-density assay was done in multiple steps using several criteria, with emphasis put on exonic SNPs while at the same time attempting a good representation of the immunome. Due to potential hairpin and dimmer artefacts, the AssayDesign software only allowed multiplexing 107 SNPs, covering 58% (104 in 180) of the polymorphic immune genes. After validation, the number of usable variation was downsized to 91 SNPs, representing only 89 immune genes (49%) but yet covering a wide array of important pathways, including IMD, RNAi, JAK/STAT. Besides, 23 of these genes might be of adaptive relevance because they are differentially expressed in bees infected with different viruses and *Nosema* spp.^17,31,33,39,40,43–45^.

The 107 selected SNPs were validated on 18 independent drones (haploid males) by comparing the SNP calls generated by the iPLEX MassARRAY system with those obtained in the Illumina HiSeq 2500 platform. This endeavour was greatly facilitated by the haplodiploid sex determination system in *A. mellifera* because, by screening drones instead of the traditionally used workers (diploid non-fertile females), we were able to detect allelic drop-in artefacts in the erroneously typed heterozygous loci. Fourteen SNPs showed heterozygous calls in > 10% of the drones, which was the main source of mismatch (14 out of 16 problematic SNPs) between the two platforms. This 13% (14 out of 107 typed SNPs) allele drop-in error was considerably higher than that obtained (2%, 3 out of 127 typed SNPs) with a SNP assay designed in a previous study for detecting introgression in *A. m. mellifera*, which was also typed with the iPLEX MassARRAY system in an independent dataset^52^. Allele drop-in could not be due to contamination because the 14 problematic SNPs were spread across the four assays and the remaining loci were correctly typed as homozygous and matched the calls of the Illumina HiSeq 2500 platform. Instead, allele drop-in could be explained by gene homology^52^, although this is an unlikely source of error because the primers designed by the AssayDesign software were all verified *in silico* for potential binding to multiple targets. Nonetheless, this hypothesis cannot be completely ruled out because the honey bee genome is not fully curated. Given that the genotyping with the two different assays was outsourced to different laboratories^52^, which have distinct MassARRAY models, the difference in the error rates could be explained by a technical artefact. After removing the problematic SNPs, the remaining 91 were accurately called in the 18 drone samples and successfully genotyped in the 156 independent samples, as reflected by the low rate of missing data (3.5%). Among the 91 quality-proved SNPs, of particular interest are 36 that result in non-synonymous changes, as these are likely to have a functional effect. However, the remaining 55 are also promising as they may affect gene regulation or expression and thus also have a functional role^64^.

Evidence from many organisms indicates that sequence variation in immune genes affects host susceptibility to infections^22,24–27^. In honey bees, the interaction between the immune system and pathogens has been addressed only through employment of gene expression assays^11,30–33^. These assays showed differential expression in immune genes when honey bees are infected with SINV^43^, IAPV^31,43^, DWV^40^ and *Nosema* spp.^33,45^. But more importantly, these assays showed differential expression between tolerant and sensitive bee strains to infections of *N. ceranae* and *V. destructor*^30,38,65^. Despite the shallow coverage of the honey bee immunome, the SNP assay developed here can be used as a very first step towards unravelling the genetic basis of honey bee susceptibility to infections through case–control association tests, similar to studies on other organisms^37,66–69^. Our assay is promising for this type of analyses for different reasons. First, although the assay only partially samples the immunome (average one SNP per gene and 89 of the 180 annotated genes), it includes SNPs located in the main pathways, such as IMD, JAK-STAT, Toll and RNAi, and there is evidence that a small number of large-effect genes have a substantial contribution to the genetic variation of host susceptibility^27–29^, increasing the probability of finding associations. Second, 82% of the SNPs lay in protein-coding regions and the non-synonymous SNPs may directly change protein function. Third, while the remaining SNPs fall outside of protein-coding regions, they are not only mapped to other relevant regions, potentially affecting transcription regulation (5’UTR), messenger RNA stability (3’UTR), and binding of the transcription initiation complex to the promoter (< 1 Kb upstream of the transcription start site^21,64^), but also in linkage with the coding region. Finally, some of the SNPs are located in genes that showed to be differentially expressed in honey bees infected with SINV^43^, IAPV^31,43^, DWV^40^ and *Nosema* spp.^33,45^.

The medium-density immune assay developed here, as well as other dedicated assays^42,52,59–62^, is not recommended for standard population genetics analyses relying on allele frequency spectrum in different genomic regions or neutrality (e.g. nucleotide diversity, population size, migration, population structure). This is because genetic inference can be distorted due to ascertainment bias^70,71^ as well as to biases introduced from choosing high-graded SNPs segregating among target populations^72^, or from targeting only functional regions^73^, which might not be neutral. Particularly, SNPs in immune genes are expected to be under selective pressure by the spread of new parasites and pathogens or by evolution of the existing ones^28,74,75^, which can act to increase the frequency of beneficial alleles or to maintain polymorphisms. Therefore, caution is warranted when using this biased subset of the honey bee genome to infer broader patterns of differentiation because, as shown for other organisms, disease-related genes may have excessive SNPs with lower or higher levels of population differentiation due to purifying^76^, balancing^77^ or directional selection^76^. Notwithstanding these biases, inclusion of non-neutral or highly informative markers in dedicated assays may be advantageous when attempting to (i) assign individuals to source populations, (ii) estimate genetic admixture or even (iii) infer structure on ecological (rather than evolutionary) time-scale (reviewed by Helyar, et al.^78^). In this study, the ADMIXTURE analysis clustered the reference samples according to their lineage and subspecies, suggesting that our medium-density assay can be used to infer population structure, consistent with findings reported for other organisms with immune SNPs^73,79^. Furthermore, the admixture patterns inferred for the 156 samples are consistent with the conservation status and the genetic background that has been documented for the populations from where they were collected. Samples originating from the *A. m. mellifera* population of Ouessant and from *A. m. iberiensis* of mainland Portugal and Spain show a high genetic integrity as well as a shared ancestry, as expected for these sister subspecies^41,80,81^. In contrast, samples from the Azorean island of São Miguel and, more prominently, samples from mainland France and Israel are severely introgressed, as a result of beekeeper-mediated processes linked to recurrent use of commercial stock typically of C-lineage ancestry^50,51,82–84^.

Here, we detailed the steps involved in developing a medium-density assay for genotyping immune-related SNPs with the iPLEX MassARRAY system. We provided the genomic information along with the PCR and iPLEX primers for the 91 quality-proved SNPs, enabling immediate application of the immune assay and analysis of the output does not require powerful computational resources or advanced bioinformatics skills. The 91 SNPs are mapped to coding regions (and some even involve amino acid changes) or in their close vicinity. Whether they are directly associated to immune response to pathogens or in linkage with causative SNPs not included in our assay requires functional or expression studies^85^. The assay was tailored for assessing diversity in immune genes and allows for high-throughput screening of samples in a time- and cost-effective manner. In addition to potential application in studies searching for associations between molecular markers and response to pathogens, the assay could be useful for delineating ancestry in honey bees.

## Methods

### Samples and whole-genome re-sequencing

The immune SNPs were identified from scanning whole genomes (WGs) previously re-sequenced for 123 individuals originating from a wide geographical area^41,42,52^ (Fig. 1; Supplementary Table S7). This WG dataset represents seven subspecies from three evolutionary lineages, with sample sizes varying from 95 for lineage M (87 *A. m. iberiensis* and 8 *A. m. mellifera*), 19 for lineage A (12 *A. m. intermissa and* 7 *A. m. sahariensis*), to 9 for lineage C (4 *A. m. ligustica*, 3 *A. m. carnica*, and 2 *A. m. siciliana*; Fig. 1). The 123 samples were run on an Illumina HiSeq 2500 platform with sequencing libraries generated using Illumina TruSeq Sample Preparation Kit (see^41^ for further details). Mapping and variant calling were performed following Henriques, et al.^41^. A total of 2,525,621 SNPs with a minor allele frequency (MAF) ≥ 0.05 were identified after the quality control step. The functional state (non-synonymous, synonymous, intronic, UTR, or intergenic) of each SNP was annotated using the reference genome Amel_4.5, the Official Gene Set 3.2 (BEEBASE) and the Entrez Gene of NCBI.

### SNP assay design

A list of 209 immune genes putatively involved in honey bee defence against viruses^14,17,54,55^ and *Nosema* spp. infections^30,33,38,45,56^ was compiled from the literature (Supplementary Table S1). These were scanned for polymorphisms in the 123 WGs, leading to detection of 35,782 SNPs located in only 180 of the 209 genes. Following application of several filters and criteria (Fig. 2), the 35,782 SNPs were narrowed down to fit the optimal plex size of 30 SNPs per assay, as recommended by Agena BioScience for the iPLEX chemistry and the MassARRAY MALDI-TOF platform (herein abbreviated to iPLEX MassARRAY). Of the three chip formats (24, 96, and 384) available for genotyping with the iPLEX MassARRAY system, we chose the 384-format because it offers the best trade-off between number of loci and number of samples. Using a medium-density SNP panel (4 assays × 30 SNPs = 120 SNPs maximum) it is possible to screen 96 individuals in one chip.

To downsize the 35,782 SNPs, while assuring that the final medium-density assay contained SNPs with functional relevance, we discarded putatively non-functional SNPs and only retained those located in (i) protein coding (synonymous and non-synonymous), (ii) intergenic < 1 Kb upstream of the transcription start site, where the promoter is expected to be located, (iii) 3’UTR, (iv) 5’UTR, and (v) intronic < 4 bp from exons, where alternative splicing occurs^86^. To further narrow down the SNP number, while assuring that the final panel contained informative SNPs for subspecies important to beekeeping in Europe (*A. m. iberiensis* and *A. m. mellifera* in lineage M; *A. m. ligustica* and *A. m. carnica* in lineage C), we discarded those with MAF ≤ 0.05 identified in a subset comprising only C- and M-lineage subspecies (Fig. 2).

The ensuing step was linked to the iPLEX MassARRAY system (Fig. 2). The 501-bp flanking regions (250 bp on either side) of all retained SNPs (3723) served as input for delineating the four assays (IA1, IA2, IA3, and IA4) with the AssayDesign Suite software (http://agenabio.com/assay-design-suite-20-software), using the default settings. The 3723 SNPS were prioritized to assure that the final panel contained the highest possible number of non-synonymous SNPs, functional SNPs with F_ST_ = 1 between M- and C- lineages (calculated in PLINK 1.9^87^), immune genes, and chromosomes. The AssayDesign Suite searched for optimal areas within the 501-bp flanking region of the 3723 SNPs for designing the PCR primers, while constructing all possible multiplex combinations. The optimal 30-SNP multiplexing capacity was attempted by the software whilst preventing hairpin and dimmer formation. In addition to the PCR primers, the software designed the iPLEX extension primers placed immediately adjacent to each SNP. The quality of each designed primer was assessed using the Primer-BLAST tool available in NCBI. This additional step in the SNP selection process allowed identification of primers matching multiple targets in the honey bee genome, and subsequent deletion of potentially problematic SNPs. Lastly, the genomic positions of the SNPs incorporated in the final panel were upgraded in the latest honey bee genome assembly Amel_HAv3.1 by using the BLASTn tool in NCBI (Supplementary Table S4).

### IPLEX MassARRAY

The four newly-designed SNP assays were genotyped using the iPLEX MassARRAY MALDI-TOF system. Briefly, the PCR primers were pooled to a final concentration of 500 nM. The PCR primer pool was used to amplify 10 ng of DNA in a 5 µl volume reaction with 1 U of FastStart Taq Polymerase (Roche, Indianapolis, IN) and 4 mM of Mg_2_Cl. PCRs were performed in a 5 µl volume using a standard 384-well plate format according to the specifications provided by Agena BioScience. The plates were cycled 45 times with an annealing temperature of 56 °C. After PCR, shrimp alkaline phosphatase (SAP) was used to dephosphorylate any remaining dNTPs to render them unusable for ensuing polymerase reactions. A total of 0.5 U of SAP and buffer (Agena Bioscience) was added to the PCR tubes and incubated for 40 min at 37 °C, followed by 5 min at 85 °C. Single base extension reactions were performed on the PCR reactions with the iPLEX Gold Kit (Agena Bioscience) and 0.8 µl of the custom UEP (unextended primer) pool. The kit contains mass-modified terminator nucleotides that increase the mass difference between extended UEPs, allowing for greater accuracy in genotyping. The mass difference with unmodified terminator nucleotides ranges from 9 to 40 kDa, depending on the two nucleotides compared. With the mass-modified terminator nucleotides the mass difference increases to 16–80 kDa. The single base extension reactions were cycled with a nested PCR protocol that used five cycles of annealing and extension nested with a denaturation step in a cycle that was repeated 40 times for a total of 200 annealing and extension steps. The goal was to extend nearly all of the UEPs. Following single base extension, the reactions were diluted with 16 µl of deionized water and with 6 ng of resin. After a 20-min. deionizing step, the reactions were dispensed onto SpectroChipArrays with a Nanodispenser (Agena Bioscience). The speed of dispensation was optimized to deliver an average of 20 nL of each reaction to a matrix pad on the SpectroChip. An Agena Bioscience Compact MassArray Spectrometer was used to perform MALDI-TOF mass spectrometry according to the iPLEX Gold Application Guide^88^. The Typer 4 software package (Agena Bioscience) was used to analyse the resulting spectra and the composition of the target bases was determined from the mass of each extended oligo.

### SNP assay assessment

Eighteen drones (4 *A. m. iberiensis*, 3 *A. m. mellifera*, 4 *A. m. ligustica*, 3 *A. m. carnica*, 4 *A. m. intermissa*) were chosen from the WG set for assessing SNP calling quality and genotyping accuracy of the newly developed IA1, IA2, IA3, and IA4 assays (Fig. 2). The DNAs of the 18 drones (haploid males) were previously isolated with a phenol-clorophorm protocol^89^ and these were genotyped herein using the four assays and the iPLEX MassARRAY system, as described above. The missing genotype data were calculated using PLINK. The SNP calls generated by the iPLEX MassARRAY system were compared with those obtained previously with the Illumina HiSeq 2500 platform^41,42,52^ for the WGS and the number of inconsistent genotypes was recorded.

### SNP assay application

The four assays were applied to 156 samples collected between 2018 and 2019 from colonies deployed in Spain (N = 34), France (N = 36), Israel (N = 40), and Portugal (N = 46; Fig. 1; Supplementary Table S6). Each of the 156 samples represented a single colony and consisted of a pool of 10 workers (diploid non-fertile females). Total DNA was extracted from each pool (20 front legs) using the Ron’s Tissue DNA Mini Kit (Bioron), following manufacturer instructions with slight modifications. The 156 samples were genotyped using the iPLEX MassARRAY system, as described above.

The allele frequencies were plotted for each SNP using the R script described in Jenkins, et al.^90^ with slights modifications. The membership proportions (Q-values) were inferred for the 156 pooled samples with ADMIXTURE^91^ for a number of K genetic groups ranging from one to seven, using 10,000 iterations in 20 independent runs. The convergence between iterations was monitored by comparing log-likelihood scores (LLS) using the default termination criterion set to stop when LLS increases by < 0.0001 between iterations. CLUMPAK^92^ was used to summarize and visualize the Q-plots. A subset of the whole-genome sequenced individuals (15 *A. m. iberiensis*, 12 *A. m. intermissa*, 4 *A. m. ligustica*, 3 *A. m. carnica*, 8 *A. m. mellifera*, 7 *A. m. sahariensis* and 2 *A. m. siciliana*) was used as a reference in the ADMIXTURE analysis. The most probable K was estimated for this dataset using the 5- fold cross-validation (CV) error.

## Supplementary Information

Supplementary Figures

Supplementary Tables
