# Supplementary material for: A SNP assay for assessing diversity in immune genes in the honey bee (Apis mellifera L.)
Source: Sci Rep. 2021 Jul 28;11:15317. doi: 10.1038/s41598-021-94833-x (PMC8319136; doi:10.1038/s41598-021-94833-x)
Supplement: Supplementary file 1 — Supplementary Figures [file 41598_2021_94833_MOESM1_ESM.docx]

**Supplementary Figures**

A SNP assay for assessing diversity in immune genes in the honey bee (*Apis mellifera* L*.*)

Dora Henriques^1^, Ana R. Lopes^1^, Nor Chejanovsky^2^, Anne Dalmon^3^, Mariano Higes^4^, Clara Jabal-Uriel^4^, Yves Le Conte^3^, Maritza Reyes-Carreño^3^, Victoria Soroker^2^, Raquel Martín-Hernández^4,5^, M. Alice Pinto^1*^

^1^Centro de Investigação de Montanha, Instituto Politécnico de Bragança, Campus de Santa Apolónia, 5300-253 Bragança, Portugal.

^2^Agricultural Research Organization, The Volcani Center, Israel.

^3^ INRAE, Unité Abeilles et Environnement, Avignon, France

^4^IRIAF. Instituto Regional de Investigación y Desarrollo Agroalimentario y Forestal, Laboratorio de Patología Apícola, Centro de Investigación Apícola y Agroambiental (CIAPA), Consejería de Agricultura de la Junta de Comunidades de Castilla-La Mancha, Marchamalo, Spain.

^5^ Instituto de Recursos Humanos para la Ciencia y la Tecnología (INCRECYT-FEDER), Fundación Parque Científico y Tecnológico de Castilla—La Mancha, 02006 Albacete, Spain

*Corresponding author:

Maria Alice Pinto

Instituto Politécnico de Bragança

5300-253 Bragança, Portugal

E-mail: [apinto@ipb.pt](mailto:apinto@ipb.pt)


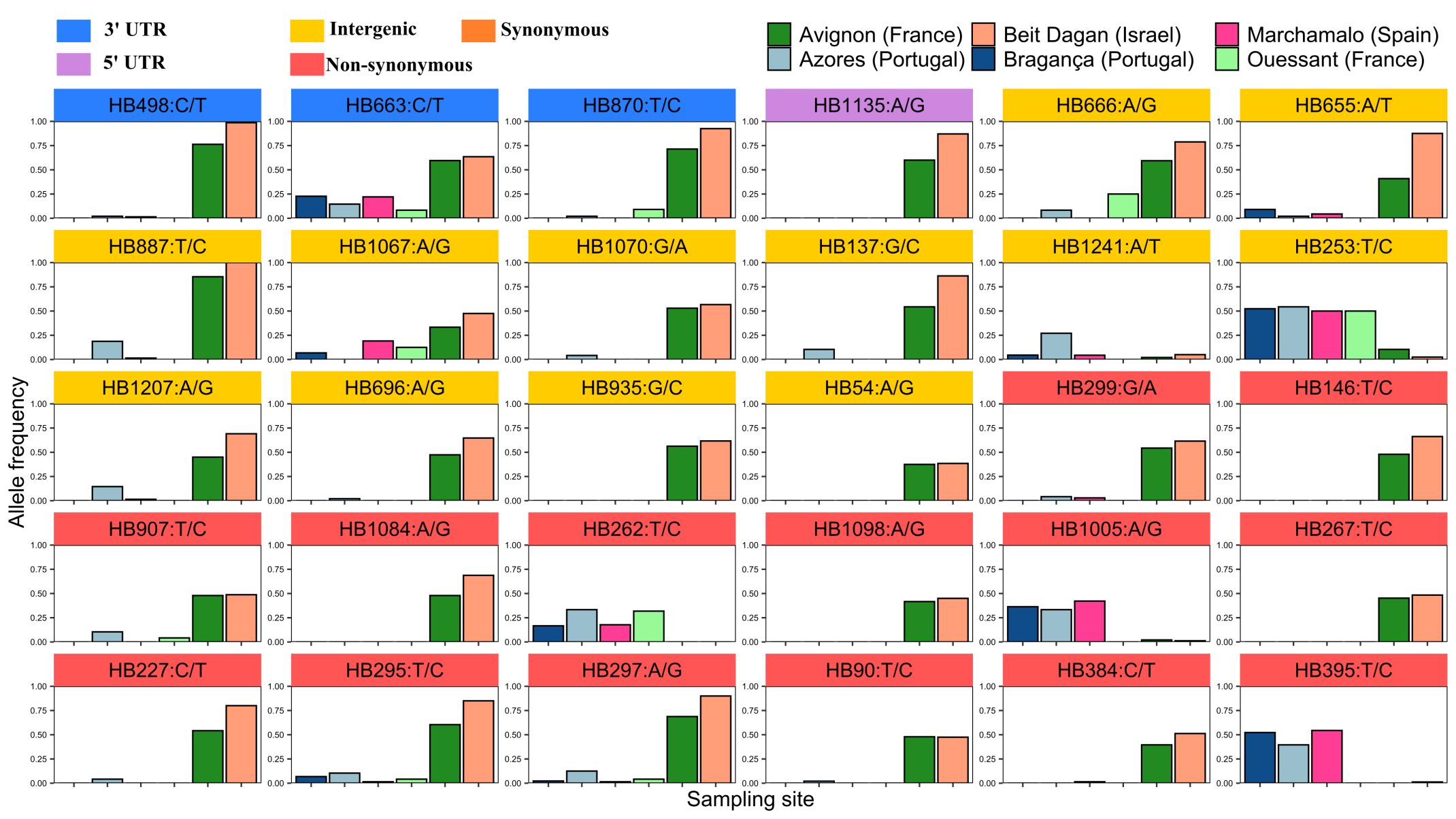


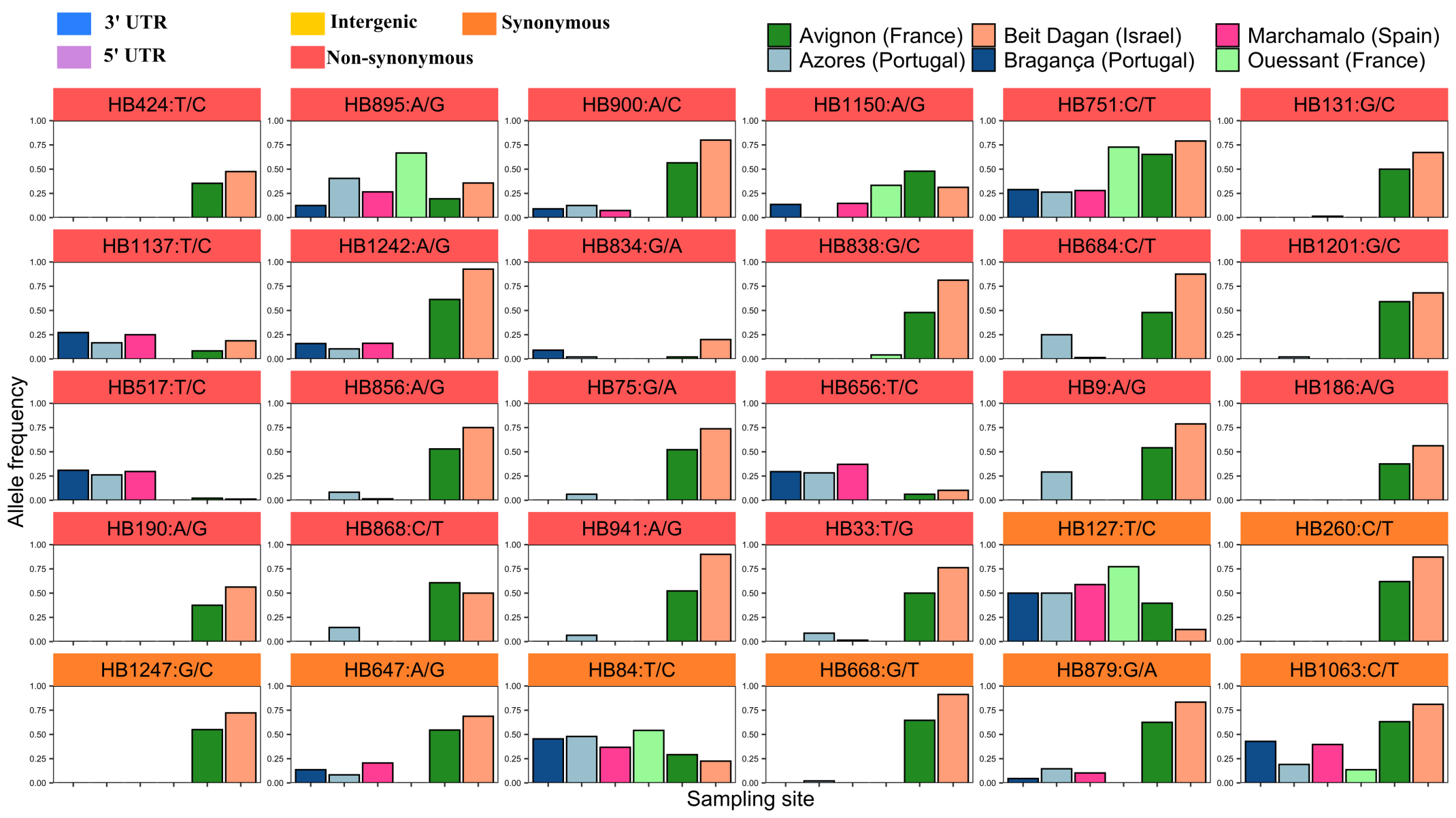


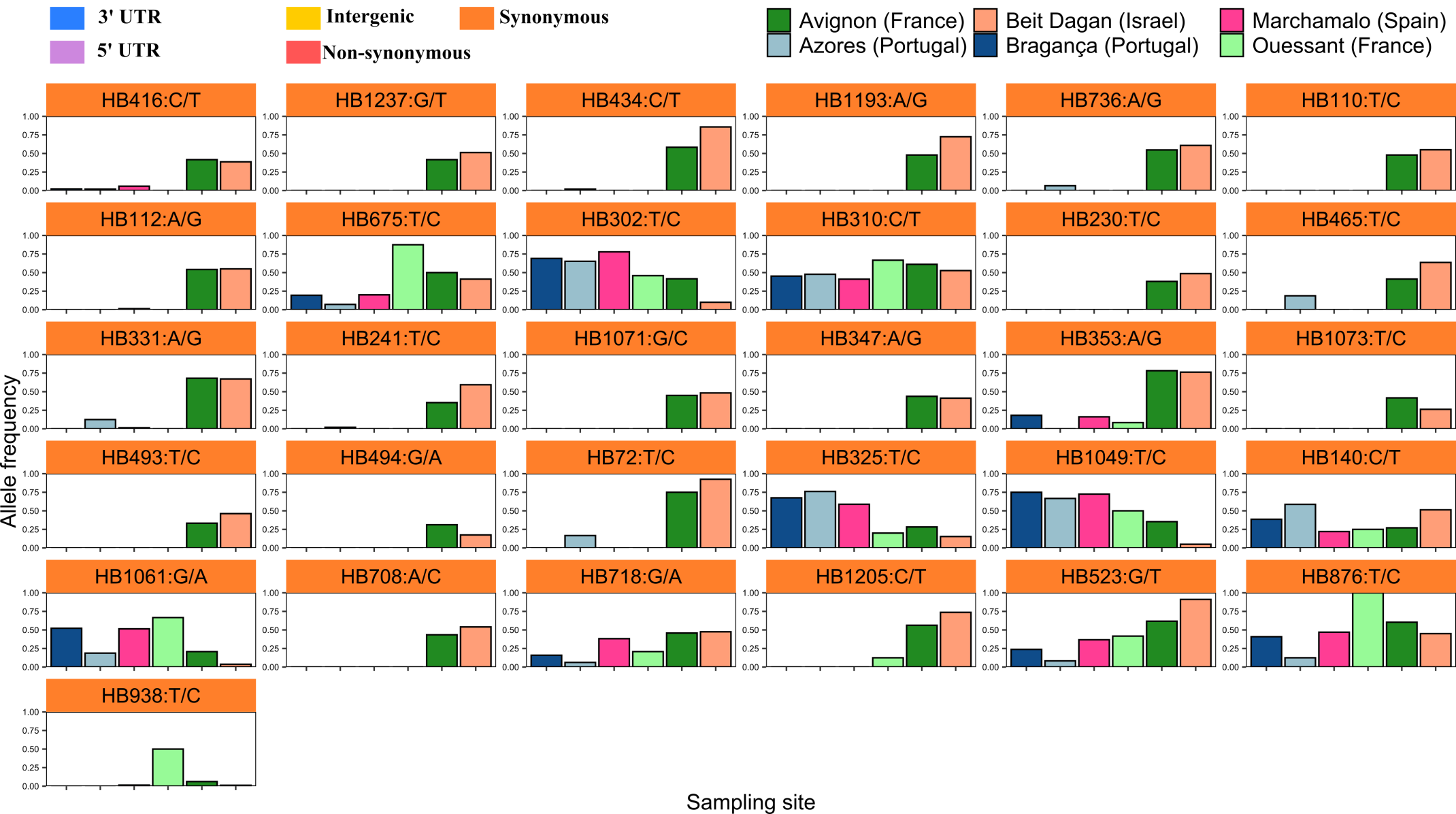


**Figure S1.** Distribution of the minor allele frequency for each SNP and population. On top of the chart, there is the SNP name (e.g. HB498) and the corresponding alleles (e.g. C/T) the first allele (e.g. C) is the minor allele. Groups of SNPs are organized by functional state.


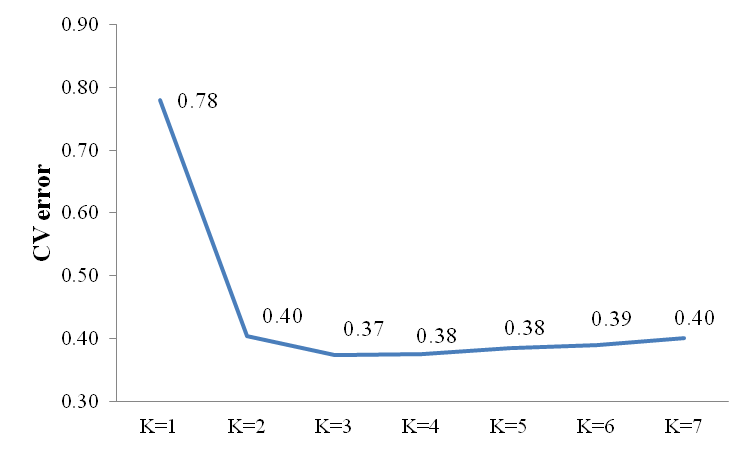


**Figure S2.** Error rate estimated by Cross-Validation (CV) test for each K (1-7) in ADMIXTURE analysis. The lowest value provides the best K (K=3).
